# Cone‐Beam CT image contrast and attenuation‐map linearity improvement (CALI) for brain stereotactic radiosurgery procedures

**DOI:** 10.1002/acm2.12477

**Published:** 2018-10-19

**Authors:** SayedMasoud Hashemi, Christopher Huynh, Arjun Sahgal, William Y. Song, Håkan Nordström, Markus Eriksson, James G. Mainprize, Young Lee, Mark Ruschin

**Affiliations:** ^1^ Department of Radiation Oncology Sunnybrook Health Sciences Centre Toronto ON Canada; ^2^ Elekta Instrument AB Stockholm Sweden; ^3^ Sunnybrook Research Institute Toronto ON Canada; ^4^ Department of Physics Ryerson University Toronto ON Canada

**Keywords:** artifact reduction, Cone‐beam CT, image‐guidance, radiosurgery

## Abstract

A Contrast and Attenuation‐map Linearity Improvement (CALI) framework is proposed for cone‐beam CT (CBCT) images used for brain stereotactic radiosurgery (SRS). The proposed framework is tailored to improve soft tissue contrast of a new point‐of‐care image‐guided SRS system that employs a challenging half cone beam geometry, but can be readily reproduced on any CBCT platform. CALI includes a pre‐ and post‐processing step. In pre‐processing we apply a shading and beam hardening artifact correction to the projections, and in post‐processing step we correct the dome/capping artifact on reconstructed images caused by the spatial variations in X‐ray energy generated by the bowtie‐filter. The shading reduction together with the beam hardening and dome artifact correction algorithms aim to improve the linearity and accuracy of the CT‐numbers (CT#). The CALI framework was evaluated using CatPhan to quantify linearity, contrast‐to‐noise (CNR), and CT# accuracy, as well as subjectively on patient images acquired on a clinical system. Linearity of the reconstructed attenuation‐map was improved from 0.80 to 0.95. The CT# mean absolute measurement error was reduced from 76.1 to 26.9 HU. The CNR of the acrylic insert in the sensitometry module was improved from 1.8 to 7.8. The resulting clinical brain images showed substantial improvements in soft tissue contrast visibility, revealing structures such as ventricles which were otherwise undetectable in the original clinical images obtained from the system. The proposed reconstruction framework also improved CT# accuracy compared to the original images acquired on the system. For frameless image‐guided SRS, improving soft tissue visibility can facilitate evaluation of MR to CBCT co‐registration. Moreover, more accurate CT# may enable the use of CBCT for daily dose delivery measurements.

## 1 INTRODUCTION

The Leksell Gamma Knife Icon (Elekta AB, Stockholm, Sweden) integrates a cone‐beam CT (CBCT) image guidance system^1^ with an irradiation unit to enable frameless stereotactic radiosurgery (SRS).^2^ The CBCT system provides the stereotactic reference, which together with co‐registration of the planning image volume and the CBCT volume gives the transformation mapping of the planned isocenter positions to stereotactic coordinates. Since the X‐ray beams cannot penetrate through the frame fixation on the treatment bed, a half cone geometry is used in the Icon system, which maximizes the field‐of‐view by aligning the X‐ray tube parallel and close to the frame plane.^3^ The half cone geometry results in more pronounced shading and cone‐beam artifacts than the regular full cone geometries used in other commercial systems.

In the context of frameless SRS, improving CBCT image quality is highly motivated since stereotactic localization is based on MRI‐to‐CBCT co‐registration. Although the commercial system uses a robust co‐registration algorithm, the ability of the clinician to visualize soft tissue around the location of the brain lesions is crucial in order to verify target location. Additionally, if improvements in CT# accuracy could be made the CBCT images could also be used to calculate the daily delivered doses. Unlike in CT where a calibration phantom is commonly used to map the measured CT# to the electron density of the tissues,^4^ in the native CBCT images this process is prohibited due to the resulting artifacts that are present. Improvement of the sources of artifacts in cone‐beam CT has been an area of active research within the past few years, with dedicated frameworks being researched to correct for: shading artifact,^5^ scatter,^6^, ^7^, ^8^ and system blur.^3^ A major area of focus of CBCT image quality improvement has been scatter reduction,^9^ with several model‐based approaches^7^, ^8^ including a dedicated cone‐beam breast CT model.^7^ For the Icon CBCT system, additional challenges such as a pronounced beam hardening, a bow‐tie filter and the presence of metal (in the case of frame‐based SRS) coupled with the half cone geometry, and limited acquisition span make it necessary to add to the robust model‐based approaches such as by Zhao et al.^8^ and propose a dedicated framework that works for the SRS system.

The purpose of the present study is to develop and evaluate a computationally efficient CBCT contrast improvement and attenuation‐map correction framework for point‐of‐care Icon CBCT images without modifying the clinical workflow, i.e., no extra calibration measurements are required. The proposed framework is tested with CatPhan phantom and clinical brain images.

## 2 METHODS

The proposed framework called CALI (Contrast and Attenuation–map Linearity Improvement) considers three important sources of the non‐linearity in CBCT images, namely scattering, beam hardening, and blurriness. To improve the spatial resolution of the CBCT images, a high spatial resolution iterative reconstruction algorithm called simultaneous deblurring and iterative reconstruction (SDIR) was proposed, which estimates the blurriness in the image domain.^3^ SDIR improves the spatial resolution, preserves edges, and improves the visibility of soft tissues in the brain.^3^ For example, some brain folds and structures, such as ventricles, become clearly visible in the images reconstructed with SDIR. However, as is common in CBCT, SDIR reconstructed images still suffer from image inhomogeneity/non‐linearity, which is mainly caused by scatter contamination and beam hardening.

The CALI framework consists of pre‐processing, iterative reconstruction, and post‐processing phases. In the pre‐processing phase, a low frequency artifact correction (LFAC) algorithm and a beam hardening correction (BHC) method are applied to the projection images. The pre‐processed projections are then reconstructed with SDIR.^3^ In the post‐processing phase, a dome artifact correction (DAC) is applied on the images reconstructed with SDIR. In the case of using fixed frames during the treatment, a metal artifact reduction (MAR) algorithm will be used with the LFAC algorithm. All of the acronyms used in the present manuscript are defined in Table 1.

**Table 1:** Summary of abbreviations used in the manuscript

Abbreviation	Description
BHC	Beam hardening correction
CALI	Contrast and attenuation‐map linearity improvement
CBCT	Cone beam computed tomography
CNR	Contrast‐to‐noise ratio
DAC	Dome artifact correction
FBP	Filtered back projection
FDK	Feldkamp‐davis‐kress (FDK) reconstruction
LFAC	Low frequency artifact correction
MAR	Metal artifact reduction
MTF	Modulation transfer function
PSF	Point spread function
SDIR	Simultaneous deblurring and iterative reconstruction
TV	Total variation

BHC plays a fundamental role in reducing CT# errors. In the CALI framework, LFAC algorithm not only improves the homogeneity of the images, but also, makes the application of the proposed BHC algorithm possible. The BHC uses an attenuation value mapping on the basis materials, which depends on the accuracy of the reconstructed attenuation values. The reconstructed attenuation‐map in CBCT images usually contains large errors, mainly due to scatter contamination and flood image errors from detector saturation. The LFAC reduces the reconstruction error, enabling the BHC algorithm to effectively use the linear attenuation mapping. After reconstructing the pre‐processed projections with SDIR, due to the application of a bowtie filter in the Icon, the images suffer from dome artifact, which should be corrected to achieve higher CT# linearity. This is done with the proposed DAC algorithm.

The only required a priori in the proposed framework is the X‐ray spectrum. In this study we used a Monte‐Carlo simulated spectrum. However, the X‐ray spectrum can also be estimated from the X‐ray projections of a known object.^10^, ^11^

### 2.A Low frequency artifact correction

**Figure 1:**
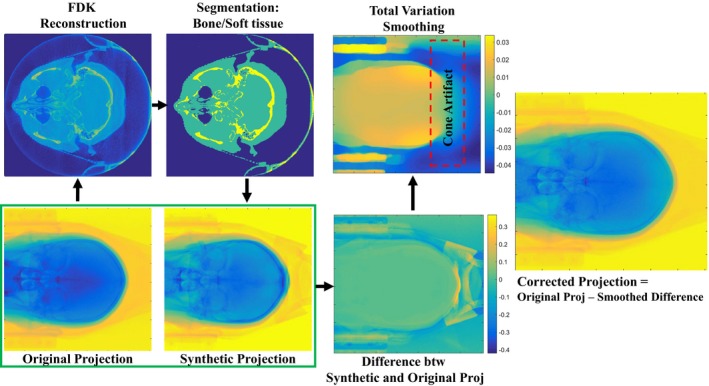
Flowchart of the low frequency artifact correction (LFAC) algorithm.

The proposed LFAC algorithm, as shown in Fig. 1, includes segmentation of the brain images reconstructed with regular filtered back projection (i.e., Feldkamp method,^12^ “FDK”) into bony and soft tissue (brain white/gray matter) regions. A thresholding is used for this segmentation task, where the threshold values are estimated from the histogram of the images.^13^ The segmentation information is fed into a polychromatic forward projector with the attenuation and mass density values of the soft tissue (brain) and bone calculated from the tables provided by NIST (https://www.nist.gov/pml/X-ray-mass-attenuation-coefficients). The forward projector is a 3‐D ray‐driven method that uses bilinear interpolation and contains two loops over material and energy, respectively, to consider the energy dependent values of the X‐ray attenuation. The X‐ray spectrum is estimated by Monte Carlo simulation of the Icon X‐ray source, using Elekta's proprietary Monte Carlo simulator (Pegasos) which uses PENELOPE at its core.^14^ This can also be done by an iterative spectrum estimation algorithm.^10^, ^11^ The difference between the measured projections and the ideally calculated projections shows variations in different regions, caused by different factors including scattered photons and the saturation of the calibration gain/flood images used to normalize the measured projections. This difference is filtered with a smoothing total variation (TV) denoizing method,^15^ to preserve the larger edges and to remove the remaining structures. The smoothed projection differences are then subtracted from the original measured projections. Reconstructing the corrected projections, it could be seen that the errors of the reconstructed attenuation map are highly reduced. As can be seen in Fig. 1, the cone‐beam artifact increases the error at the top of the head. To determine the region affected by the cone artifact, we first set all the voxels in the entire image volume to one. We subsequently forward and back project the unity image volume and threshold voxels that differ from one by more than 30%. Those voxels define the cone‐artifact region that we subsequently interpolate. We used a TV in‐painting algorithm for interpolation as it is robust to error and noise in neighboring pixels.^16^ Since the LFAC involves forward projecting the image, the artifact propagates into the synthetic projections and would be back‐projected into the image domain introducing additional streak artifacts. Therefore, in LFAC the removal and subsequent interpolation prevents propagation of that cone artifact error to the next step.

### 2.B Beam hardening correction

The output of LFAC is fed into a BHC algorithm. This BHC algorithm is similar to previously described work,^17^ but, instead of utilizing segmented tissues, the reconstructed attenuation value of the pixels are linearly mapped onto *M* basis materials using a linear mapping function as below, in which $\mu_{i},i=1,\ldots ,M$ denotes the *M* basis materials:

(1) $$T_{i}f\left(x\right)=\left\{\begin{array}{cc} \frac{\mu_{i}-f\left(x\right)}{\mu_{i}-\mu_{i-1}}\mu_{i-1} & \mu_{i-1}<f\left(x\right)<\mu_{i}\\ \frac{f\left(x\right)-\mu_{i}}{\mu_{i+1}-\mu_{i}}\mu_{i} & \mu_{i}<f\left(x\right)<\mu_{i+1} \end{array}\right.$$

where *f(x)* is the reconstructed attenuation map. Note that since the pixel values larger than basis material attenuation values are not possible using the basis materials, we try to choose materials with larger attenuation as any one of the basis materials and the larger values are capped. This would be corrected in subsequent iterations. Three materials are used in this paper, namely bone, soft tissue, and air. Then, the measured projections will be corrected by iteratively adding the difference between the simulated monochromatic (i.e., $P_{\mathrm{Mono}}^{\left(n\right)}=\sum_{i=1}^{M}g_{n,i}$ and $g_{n,i}=PT_{i}f_{n}$ for *i* ^th^ basis material *n* ^th^ iteration with *P* being the forward cone beam X‐ray projection) and polychromatic projections calculated from the mapped basis materials $P_{\mathrm{poly}}^{\left(n\right)}=-log\left(\int \hat{S}\left(E,x\right)e^{-\sum_{i=1}^{M}\hat{\sigma }\left(E,x\right)g_{n,i}}dE\right)$, with $\hat{\sigma }\left(E,x\right)$ being the ratio between the attenuation of the basis materials in different energy bins and the equivalent attenuation at the mean energy, calculated by $\int \hat{S}\left(E,x\right)\mu \left(E,x\right)dE$, and $\hat{S}\left(E\right)=\frac{S(E)}{\int S(E)dE}$ is the normalized spectrum of the Icon X‐ray tube. Using these notations the updated projections at the (*n*+1)^th^ iteration would be:

(2) $$P^{\left(n+1\right)}=-\log\left(I_{C}\right)+\left(P_{\mathrm{Mono}}^{\left(n\right)}-P_{\mathrm{Poly}}^{\left(n\right)}\right)$$

where I_C_ is the output of the shading correction. The results of preliminary experiments indicated that three iterations are enough to achieve adequate BHC results, after which the difference between $\left(I_{C}-P_{\mathrm{Poly}}^{\left(n\right)}\right)$, becomes sufficiently small.

### 2.C Dome artifact correction

Because of the use of bowtie filter in Icon's CBCT system, the X‐ray spectrum has different energy distribution across the detector, which affects the attenuation values at each pixel of the image. Therefore, after the BHC step, the attenuation value of the reconstructed images are larger at the center of the image than the pixels with the same material content but away from the center. Dome/capping artifact decreases the detectability of the objects at the center of the image. To correct the dome artifact, we use the linear mapping presented in Eq. (1) where the attenuation coefficients, *μ*_i_, represent the mean attenuation of the basis materials for the spectrum energy sensed by that pixels of the image. The average energy in each voxel is estimated by energy‐dependent back‐projection of the X‐ray spectrum (similar to FDK without filtration). This contains the energy variations in the peripheral and central parts and the effect of the bowtie filter. Since the exact shape of the bowtie filter is unknown, doing the correction in the projection domain is challenging. The value of the dome artifact corrected image will be the linear combination of the attenuation value of the basis materials at a global fixed mean energy (i.e., a chosen fixed energy equal to the mean energy of the spectrum) with weights calculated from the linear map function. It should be noted that this is a post‐processing step, applied on the images reconstructed from the pre‐processed projections.

### 2.D Metal artifact correction

**Figure 2:**
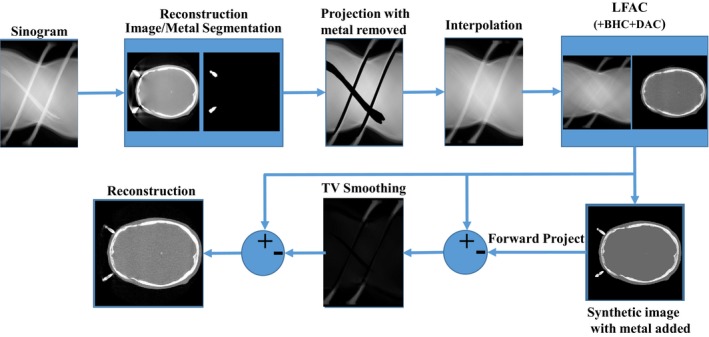
The proposed metal artifact correction flowchart.

Although the main goal of Icon CBCT system is the application of frameless planning and treatments, some of the patients are still treated using the fixed frames. This causes the familiar metal artifacts in the images. To reduce the metal artifacts in these cases the following algorithms are proposed. The projections are first processed with a method similar to “MAR” to remove the effect of metal.^18^ In this step the images are reconstructed with FDK and a threshold is applied to identify the metals in the image.^13^ The metal images are forward projected and used to mask the projections affected by metal. The masked projections are replaced with data interpolated from its neighbor's values using the aforementioned in‐painting algorithm.^16^ The proposed CALI framework is then applied on the processed projections. Then, to add the metal information, a synthetic image is built by adding the masked metal information to the CALI corrected images. In this synthetic image, the soft tissue parts of the image are replaced with a mean value calculated from all soft tissue pixels, but, all other structures are kept unchanged. This reduces the chance of affecting the low contrast structures in the soft tissue. The synthetic image is forward projected and is subtracted from the LFAC corrected projections. This difference is smoothed with TV and is added to the LFAC corrected projection. The workflow of this correction is depicted in Fig. 2.

### 2.E Experimental studies

Icon uses a 780 × 720 pixel flat panel detector, with resolution of 0.368 × 0.368 mm^2^. In all scans 334 projections are acquired from about 200° rotation around the phantom or patients. The reconstructed images have 448 × 448 × 448 voxels with size of 0.5 × 0.5 × 0.5 mm^3^. The images used in this paper include a CatPhan 503 (The Phantom Laboratory, Inc., Salem, NY, USA) scanned in Elekta (Stockholm, Sweden) at 90 kVp and 25 mA × 40 ms projections, and 11 clinical SRS images scanned in University Hospital La Timone (Marseille, France), at 90 kVp and 10 mA × 40 ms projections. It should be noted that the source of clinical images used in the present study was the same source as the study described in previous SDIR work.^3^

To accelerate the reconstruction, all methods are implemented in‐house with CUDA to run on an NVIDIA GPU (GTX970, CUDA7.5), and are compiled with Matlab as MEX files. The natively reconstructed images from the Icon system were generated using filtered backprojection (FBP) with a standard FDK approach^12^ and are denoted as “FDK” throughout the Results section. For more details on the reconstruction implementation, the readers are referred to previous SDIR work.^3^

## 3 RESULTS

**Figure 3:**
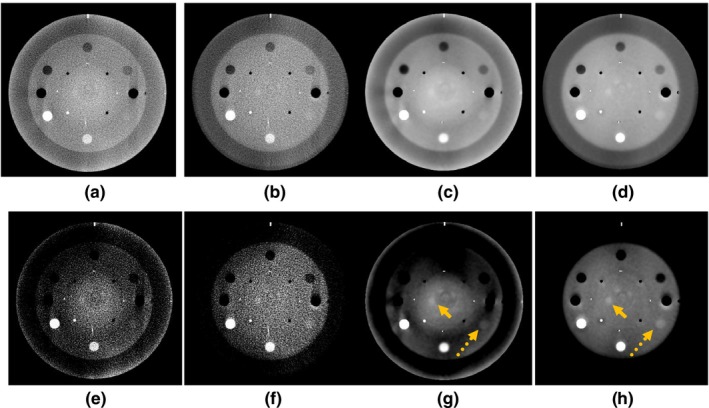
CatPhan phantom reconstructed with and without the proposed correction framework. (A, E) FDK without correction, (B,F) FDK with correction, (C,G) Simultaneous Deblurring Iterative Reconstruction (SDIR) without correction, and (d,h) SDIR with corrections. (A–D) shown with wide window/level (W = 2500, L = 600) and (E–H) shown with narrow window/level (W = 100, L = 50).

Figure 3 depicts the effect of the proposed framework on the sensitometry slice of the CatPhan phantom. An accurate CAD model of the phantom, designed based on the information provided in CatPhan 503 manual, is used in this test. As shown in Fig. 3, the contrast of the low contrast acrylic rod (illustrate by dashed arrows) is highly improved and the acrylic spheres (illustrate by solid arrows) became visible after the utilization of the proposed corrections. Defining a circular ROI in the acrylic rod with ¾ radius of the rod and an ROI in the background of the sensitometry slice, the contrast to noise ratio (CNR) of the acrylic rod was improved from 1.8 in the image reconstructed by SDIR without corrections to 7.8 in the image reconstructed by CALI framework.

**Figure 4:**
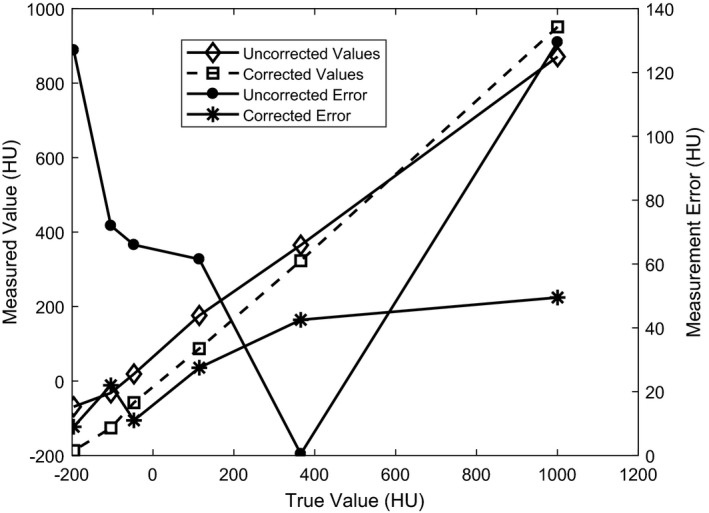
CT# linearity of the sensitometry module in CatPhan.

Representing the true CT#'s by the mean value of the true expected values, the mean absolute measurement error is reduced from 76.1 to 26.9 and the linearity (slope of the regression line) was improved from 0.80 to 0.95, as shown in Fig. 4. As can be seen in this figure, since the resulting CT# error profile of the images reconstructed with CALI is relatively constant with respect to true CT#, renormalization of the images to yield true CT# is feasible with CALI.

**Figure 5:**
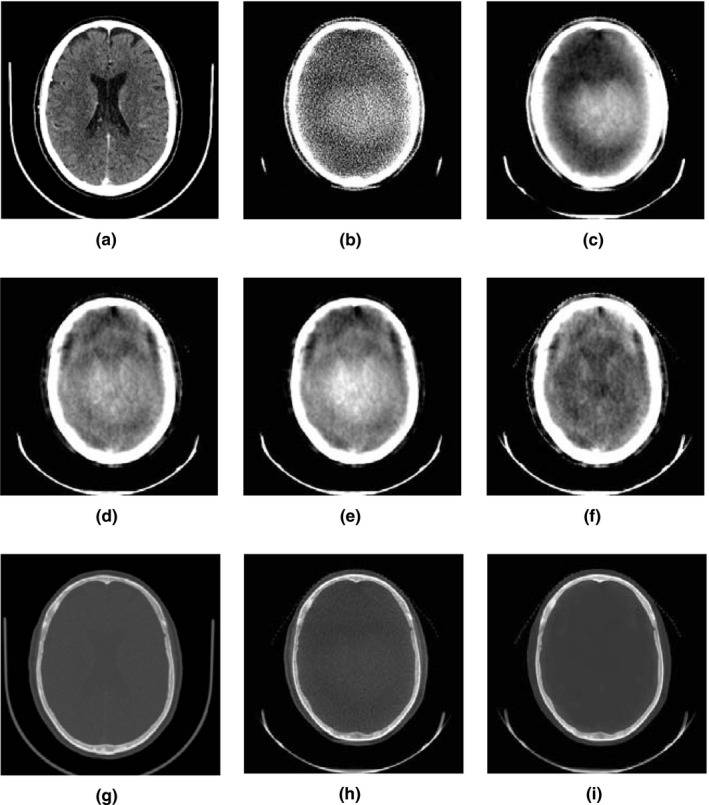
FDK and SDIR comparison with/without the proposed corrections of a patient. (A) CT image shown as the ground truth for corrected CBCT images, (B) FDK reconstructed image without correction, (C) IR without correction, (D) SDIR images with LFAC, (E) SDIR + LFAC+BHC, (F) SDIR + LFAC+BHC+DAC. Note the improvement in image uniformity in (F) compared to (E) is attributable to the addition of the dome artifact correction (DAC). Panels (A) through (F) use a narrow “soft tissue” window (W = 100, L = 50) to highlight the visibility of soft tissue structures such as ventricles. (G–I) show the CT, FDK and SDIR+CALI images on a bone window with some soft tissue presence (W = 2500, L = 600).

Figure 5 shows the effect of each pre‐ and post‐processing step on one slice of a clinical SRS case CBCT images. The images are reconstructed with FDK, and SDIR without correction, with LFAC, with LFAC+BHC, with LFAC+BHC+DAC. The same slice of the clinical CT images is provided to be compared with the CBCT images. The SDIR reconstruction results show inhomogeneity in the reconstructed attenuation values when no correction is used. However, after processing the projections with the LFAC, this inhomogeneity is reduced. As expected, after BHC, the image has higher attenuation values at the center (E), so called the dome artifact. As shown in Fig. 5, panel F, the dome artifact is corrected with the proposed DAC algorithm, which improves the contrast and homogeneity of the image.

**Figure 6:**
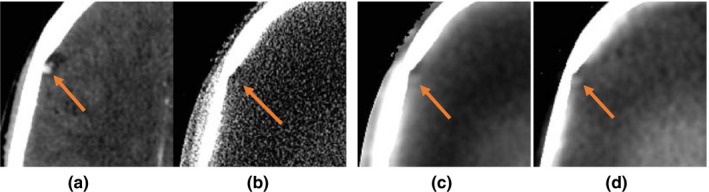
Comparing the visibility of a vessel in brain, shown with the arrow. (A) CT image shown as the ground truth for corrected CBCT images, (B) clinical CBCT image, (C) SDIR image without correction, and (D) image reconstructed with the proposed framework (CALI). The window/level was adjusted for soft tissue contrast (W = 100, L = 50).

Figure 6 shows improvements achieved in contrast detectability of a vessel in the brain. The vessel is not visible in the clinical CBCT image reconstructed with FDK. It becomes visible in the SDIR image with very low contrast. Using the proposed framework the contrast of the vessel is improved, making it more visible relative to panel B (i.e., FDK). It should be noted that the narrow window setting was manually determined for this figure to enhance the soft tissue visibility within the brain. The bone and neighboring voxels appear saturated (bright) as a result, and the narrow stretch of skin beyond the bone appears dark. Although we quote the window‐level setting used for the CT, we manually adjusted the window‐level for each CBCT scan to match that of the fan‐beam CT. Since the distribution of Hounsfield Units differ in CBCT scans, in particular for (B, FBP only) the exact window‐level setting value may be slightly different.

**Figure 7:**
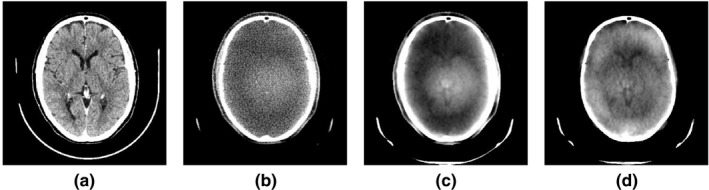
Comparing the visibility of ventricles and homogeneity improvement. (A) CT image shown as the ground truth for corrected CBCT images, (B) clinical CBCT image, (C) image reconstructed with SDIR, and (D) image reconstructed with the proposed framework. The window/levels are adjusted to maximize the soft tissue contrast in each image, (W = 100, L = 50).

Figure 7 shows the improvements achieved in the visibility and contrast of the ventricles in the images reconstructed with SDIR and the proposed framework. It is difficult to recognize the ventricles from the clinical FDK reconstruction. The iterative reconstruction improves the visibility of the ventricles, but the image includes inhomogeneity. The homogeneity and the visibility of the soft tissue details have improved significantly with the proposed framework. Note the dark ring in Fig. 7, panel D, is a result of the limitations of the DAC to perfectly correct the dome artifact. The main point is that in addition to an improvement over FDK (part B) the DAC also improves over part C, without it.

**Figure 8:**
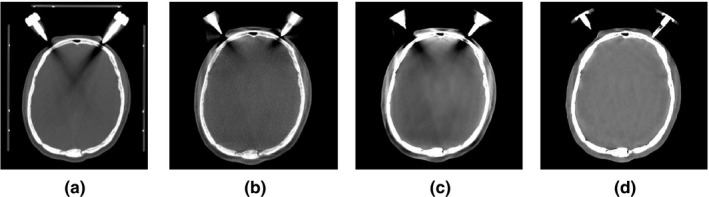
Results of metal artifact correction used together with the proposed framework. (A) CT images shown as the ground truth for corrected CBCT images, (B) clinical CBCT images, (C) SDIR reconstructed images, and (d) images reconstructed with the proposed framework including metal artifact correction.

Figure 8 shows the effect of using the proposed framework with metal artifact correction. As shown in Panels A and B, the fan‐beam CT and native FBP‐produced CBCT, respectively, suffer pronounced artifacts. The previously proposed SDIR iterative approach retains the metal‐induced artifacts (panel C) whereas the proposed CALI framework includes a step to identify the metal components and yields images (panel D) where the streaks are substantially diminished with minimal subjectively determined disruption to the nearby soft tissue structures.

## 4 DISCUSSION

A CBCT image reconstruction framework was presented to increase the homogeneity of the images and to reduce the attenuation‐map error, which in turn decreases the CT# error. The proposed method included a pre‐processing step applied on the projection images, followed by a high spatial resolution iterative reconstruction method,^3^ and post‐processing the reconstructed image with a dome artifact reduction technique. The proposed framework, CALI, was applied to a new point‐of‐care image‐guided SRS system, the Leksell Gamma Knife Icon (Elekta AB, Stockholm, Sweden).

Improving image quality of CBCT continues to be an area of active development, in particular for intraoperative imaging in image‐guided interventions.^19^, ^20^, ^21^ Improved image quality could increase utility of such systems by facilitating soft tissue surgeries, or enabling repeat imaging at sufficiently low dose, and extending applications to minimally invasive pediatric surgery.^19^ In the present manuscript, we have demonstrated that the proposed CALI framework adds to the line of CBCT image quality improvement methods currently available. Additionally, CALI may be useful in situations requiring the use of injected contrast agent in order to improve the visibility of soft tissue structures such vessels and ventricles.

It is worth noting that our LFAC workflow (Fig. 1) follows a similar workflow to that present by Zhao et al., who developed a robust scatter correction model.^8^ It should also be noted that the idea of the proposed LFAC algorithm is similar to a shading reduction method proposed by Wu et al. although their shading reduction is applied on the reconstructed images whereas we apply corrections on the projections.^5^ Due to the particulars of the Icon CBCT system's geometry and construction (compact, half cone, half‐scan, pronounced bow‐tie filter) the output of the LFAC must subsequently undergo further beam hardening correct (BHC) and our so‐called dome‐artifact correction (DAC) in order to truly realize improved image homogeneity. As noted in the Methods, our BHC is similar to the method proposed by van Gompel et al.,^17^ so it worth noting that the overall CALI framework assembles elements individually similar to published methods, but with modifications and tailoring to our point‐of‐care system. The half cone geometry used for the Icon system has previously been used in breast 3‐D X‐ray imaging but is a new geometry for this application with a larger field of view.

Despite the unfavorable geometry the proposed method showed substantial improvement in visibility of low contrast details and the homogeneity of the images. For instance, the visibility of the ventricles in the brain images and the low contrast acrylic inserts in CatPhan phantom were improved. Our measured CNR values for the acrylic insert were comparable to those found in the literature for modern and advanced CBCT systems. For example, Stankovic et al^22^ report that the native CNR for the acrylic insert on a commercial system is approximately two compared to 1.8 (for Icon FDK), which can be improved by a factor of up to five compared to our factor of four using a combination of software and hardware corrections proposed by the authors. Similarly, the CT# linearity and CT# accuracy were improved in the sensitometry module of the CatPhan phantom and were comparable to CT# linearity and integrity for head and neck sized phantoms.^22^ The attenuation‐map of the images reconstructed from shading corrected projections (i.e., processed with LFAC) were very similar to the image processed with LFAC, BHC, and DAC all together. This is due to the fact that the bowtie filter reduces the effect of the beam hardening.^23^ As a result, the LFAC processed image could be good enough for many applications, especially if very accurate CT#'s are not needed.

Major limitations of the proposed framework include the fact that the detector energy response, as well as lag and system non‐linearity, is not explicitly accounted for. Incorporation of detector response into the framework would improve the artifact corrections, as shown by work done by Sisniega et al, who developed their method and tested it on phantoms on a bench‐top system.^24^ Although the work done in Sisniega et al showed excellent artifact correction capability, accurate characterization of detector lag and line spread function were required, which are challenging to measure on clinical systems such as the Icon. Another limitation is that the framework is highly tailored toward the specific CBCT system attached to the GammaKnife Icon. A major advantage of the proposed artifact correction algorithms is that they are described step‐by‐step with enough detail to make them reproducible on other CBCT systems. The corrections need simple phantom measurements, which can all be done using the widely available CatPhan phantom. The high spatial resolution reconstruction needs a point spread function (PSF) estimation that uses the MTF module of the CatPhan phantom. The shading and BHC algorithms require the X‐ray spectrum. Although we have used Monte‐Carlo in the present work, the spectrum can also be estimated from phantom measurements using spectrum‐based algorithms.^10^, ^11^

Although the CALI component is relatively fast, the SDIR algorithm is computationally expensive, as we discuss in our previous work.^3^ We have implemented SDIR on GPU to reduce calculation time of this component. Regarding image artifacts, although the SDIR+CALI corrected images include residual dome and beam hardening artficats, the main point is that the inclusion of CALI reduces these artifacts with respect to FDK or SDIR‐only, as shown in Fig. 4 with respect to linearity. With respect to registration with MRI, the accuracy of mutual information (used in Gamma Knife) is unaffected by the window‐level used for either modality. The key issue is in how a clinician assesses the resulting co‐registration and there are two broad approaches for this: (a) using a bone window for CBCT or (b) using a narrower soft tissue window. If we use a bone window for our CBCT images the skull width is well‐visualized and co‐registration assessment involves aligning skull edges between the two modalities. If using a soft tissue window, the bone and adjacent tissue become saturated and appears thicker (even though it is not), but the evaluation of the co‐registration would focus on soft tissue areas such as the ventricles. We have ongoing work investigating the co‐registration of MRI with various CBCT algorithms. Although outside the scope of the present manuscript to comment on, the production of CBCT images with improved soft tissue CNR and uniformity will only benefit clinicians in their ability to assess co‐registration quality.

## 5 CONCLUSION

Despite the challenging CBCT geometry of the Icon commercial system, we have demonstrated that the proposed CALI method improves image quality and CT# accuracy. The CALI framework can readily be adopted to other CBCT systems, which is the scope of future work by our group. In the context of frameless SRS, the image quality improvements afforded by CALI can facilitate evaluation of MRI‐to‐CBCT image co‐registration, as well as dose calculations using daily CBCT.

## CONFLICTS OF INTEREST

Dr Sahgal has been an advisor for Abbvie; received honoraria for past educational seminars with Elekta AB, Accuray Inc., Varian Medical Systems, and BrainLAB; holds research grants with Elekta AB; and has had travel accommodations/expenses paid by Elekta AB, Varian, and BrainLAB. Dr Sahgal also belongs to the Elekta MR Linac Research Consortium. Dr Ruschin is a co‐inventor of and owns associate intellectual property specific to the image‐guidance system on the Gamma Knife Icon. Mr Eriksson and Dr Nordström are employees of Elekta Instrument AB. The other authors have no personal, financial, or institutional interest in any of the drugs, materials, or devices described in this article.

## References

[1] Jaffray DA , Siewerdsen JH , Wong JW , Martinez AA . Flat‐panel cone‐beam computed tomography for image‐guided radiation therapy. Int J Radiat Oncol Biol Phys. 2002;53:1337–1349. 12128137 10.1016/s0360-3016(02)02884-5

[2] Ruschin M , Komljenovic PT , Ansell S , et al. Cone beam computed tomography image guidance system for a dedicated intracranial radiosurgery treatment unit. Int J Radiat Oncol Biol Phys. 2013;85:243–250. 22560556 10.1016/j.ijrobp.2012.03.022

[3] Hashemi S , Song WY , Sahgal A , et al. Simultaneous deblurring and iterative reconstruction of CBCT for image guided brain radiosurgery. Phys Med Biol. 2017;62:2521–2541. 28248652 10.1088/1361-6560/aa5ed2

[4] Kanematsu N , Inaniwa T , Nakao M . Modeling of body tissues for Monte Carlo simulation of radiotherapy treatments planned with conventional x‐ray CT systems. Phys Med Biol. 2016;61:5037–5050. 27300449 10.1088/0031-9155/61/13/5037

[5] Wu P , Sun X , Hu H , et al. Iterative CT shading correction with no prior information. Phys Med Biol. 2015;60:8437–8455. 26464343 10.1088/0031-9155/60/21/8437

[6] Bootsma GJ , Verhaegen F , Jaffray DA . Efficient scatter distribution estimation and correction in CBCT using concurrent Monte Carlo fitting. Med Phys. 2015;42:54–68. 25563247 10.1118/1.4903260

[7] Shi L , Vedantham S , Karellas A , Zhu L . X‐ray scatter correction for dedicated cone beam breast CT using a forward‐projection model. Med Phys. 2017;44:2312–2320. 28295375 10.1002/mp.12213 PMC5994348

[8] Zhao W , Vernekohl D , Zhu J , Wang L , Xing L . A model‐based scatter artifacts correction for cone beam CT. Med Phys. 2016;43:1736. 27036571 10.1118/1.4943796 PMC4798999

[9] Rührnschopf EP , Klingenbeck K . A general framework and review of scatter correction methods in x‐ray cone‐beam computerized tomography. Part 1: scatter compensation approaches. Med Phys 2011;38:4296–4311. 21859031 10.1118/1.3599033

[10] Duan X , Wang J , Yu L , Leng S , McCollough CH . CT scanner x‐ray spectrum estimation from transmission measurements. Med Phys. 2011;38:993–997. 21452736 10.1118/1.3547718 PMC3041810

[11] Waggener RG , Blough MM , Terry JA , et al. X‐ray spectra estimation using attenuation measurements from 25 kVp to 18 MV. Med Phys. 1999;26:1269–1278. 10435529 10.1118/1.598622

[12] Feldkamp LA , Davis LC , Kress JW . Practical cone‐beam algorithm. J Opt Soc Am. 1984;1:612–619.

[13] Otsu N . A threshold selection method from gray‐level histograms. IEEE Trans Sys Man Cyber. 1979;9:62–66.

[14] PENELOPE2014 . A code system for Monte‐Carlo simulation of electron and photon transport. http://wwwoecd-neaorg/tools/abstract/detail/NEA-1525. Accessed March, 2014.

[15] Goldstein T , Osher S . The split bregman method for L1‐regularized problems. SIAM J Imaging Sci. 2009;2:323–343.

[16] Patel P , Prajapati A , Mishra S . Review of different inpainting algorithms. Int J Comput Appl. 2012;59:30–34.

[17] Van Gompel G , Van Slambrouck K , Defrise M , et al. Iterative correction of beam hardening artifacts in CT. Med Phys. 2011;38(Suppl 1):S36. 21978116 10.1118/1.3577758

[18] Jaiswal SP , Ha S , Mueller K . MADR‐metal artifact detection and reduction. in SPIE 9783, Medical Imaging 2016: Physics of Medical Imaging. 2016;9783:978333.

[19] Uneri A , Zhang X , Yi T , et al. Image quality and dose characteristics for an O‐arm intraoperative imaging system with model‐based image reconstruction. Med Phys. 2018; [Epub ahead of print]. 10.1002/mp.13167 PMC6711149 30180274

[20] Liu WP , Reaugamornrat S , Deguet A , et al. Toward intraoperative image‐guided transoral robotic surgery. J Robot Surg. 2013;7:217–225. 25525474 10.1007/s11701-013-0420-5 PMC4267258

[21] Guerrero ME , Jacobs R , Loubele M , Schutyser F , Suetens P , van Steenberghe D . State‐of‐the‐art on cone beam CT imaging for preoperative planning of implant placement. Clin Oral Investig. 2006;10:1–7. 10.1007/s00784-005-0031-2 16482455

[22] Stankovic U , van Herk M , Ploeger LS , Sonke JJ . Improved image quality of cone beam CT scans for radiotherapy image guidance using fiber‐interspaced antiscatter grid. Med Phys. 2014;41:061910. 24877821 10.1118/1.4875978

[23] Mail N , Moseley DJ , Siewerdsen JH , Jaffray DA . The influence of bowtie filtration on cone‐beam CT image quality. Med Phys. 2009;36:22–32. 19235370 10.1118/1.3017470

[24] Sisniega A , Zbijewski W , Xu J , et al. High‐fidelity artifact correction for cone‐beam CT imaging of the brain. Phys Med Biol. 2015;60:1415–1439. 25611041 10.1088/0031-9155/60/4/1415

